# The *Phlebotomus papatasi* systemic transcriptional response to trypanosomatid-contaminated blood does not differ from the non-infected blood meal

**DOI:** 10.1186/s13071-020-04498-0

**Published:** 2021-01-06

**Authors:** Megan A. Sloan, Jovana Sadlova, Tereza Lestinova, Mandy J. Sanders, James A. Cotton, Petr Volf, Petros Ligoxygakis

**Affiliations:** 1 Department of Biochemistry, University of Oxford, South Parks Rd, Oxford, OX1 3QU UK; 2 The Wellcome Sanger Institute, Wellcome Genome Campus, Hinxton, CB10 1SA Cambridgeshire UK; 3 Department of Parasitology, Faculty of Science, Charles University, Prague, Czech Republic

## Abstract

**Background:**

Leishmaniasis, caused by parasites of the genus *Leishmania*, is a disease that affects up to 8 million people worldwide. Parasites are transmitted to human and animal hosts through the bite of an infected sand fly. Novel strategies for disease control require a better understanding of the key step for transmission, namely the establishment of infection inside the fly.

**Methods:**

The aim of this work was to identify sand fly systemic transcriptomic signatures associated with *Leishmania* infection. We used next generation sequencing to describe the transcriptome of whole *Phlebotomus papatasi* sand flies when fed with blood alone (control) or with blood containing one of three trypanosomatids: *Leishmania major*, *L. donovani* and *Herpetomonas muscarum*, the latter being a parasite not transmitted to humans.

**Results:**

Of the trypanosomatids studied, only *L. major* was able to successfully establish an infection in the host *P. papatasi*. However, the transcriptional signatures observed after each parasite-contaminated blood meal were not specific to success or failure of a specific infection and they did not differ from each other. The transcriptional signatures were also indistinguishable after a non-contaminated blood meal.

**Conclusions:**

The results imply that sand flies perceive *Leishmania* as just one feature of their microbiome landscape and that any strategy to tackle transmission should focus on the response towards the blood meal rather than parasite establishment. Alternatively, *Leishmania* could suppress host responses. These results will generate new thinking around the concept of stopping transmission by controlling the parasite inside the insect.
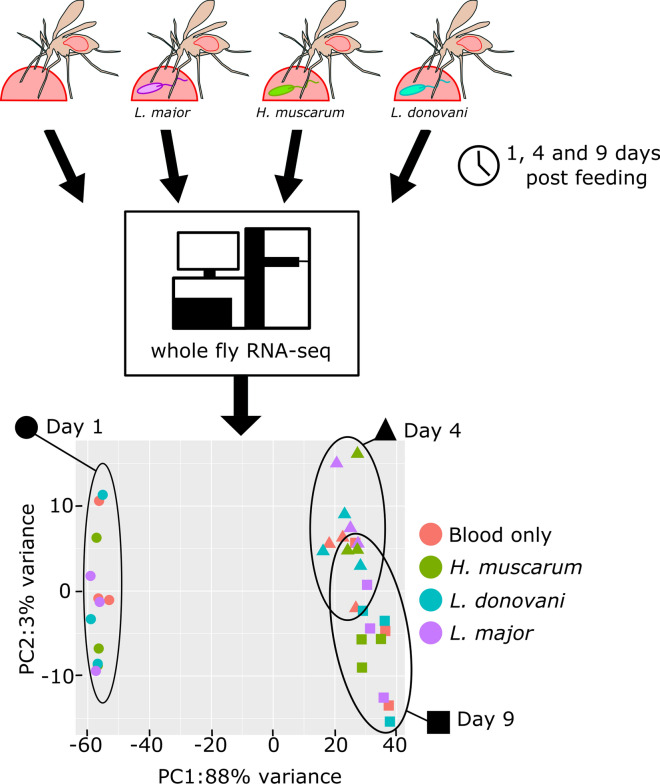

## Introduction

Leishmaniasis, a disease caused by parasites of the genus *Leishmania*, is endemic in 85 territories across the globe, with more than 600,000 cases worldwide and a prevalence of 4,000,000 [1].* Leishmania* spp. infect vertebrates through the bite of an infected sand fly vector (Diptera: Phlebotominae). The acute form of disease, visceral leishmaniasis (VL), or kala-azar, is fatal in 95% of untreated cases, with up to 50,000 people dying annually though non-fatal infections causing dermatological symptoms (cutaneous leishmaniasis), the most common consequence of infection [1]. Although the ongoing VL elimination program in the Indian subcontinent is proving successful against the most severe clinical forms of VL [2], the elimination of leishmaniasis will likely require a combination of transmission-blocking strategies and novel treatments. This is especially the case in light of reports of resistance to drugs used to treat human infections [3, 4] as well as resistance to the pesticides used to control vector populations [5–7]. However, to develop approaches to blocking transmission, a better understanding is needed of the basic biology that underlines the interactions between parasite and insect vector.

The sand fly responses to blood-feeding have been investigated, with several gene families shown to be transcribed and/or expressed in response to a blood meal [8], including those encoding digestive enzymes, such as trypsins and chymotrypsins, pathogen recognition molecules and components of the peritrophic matrix, a protective chitinous mesh which lines the midgut after ingestion [8]. However, few sand fly genes or transcripts specifically associated with *Leishmania* infection have been identified. There is some evidence to suggest that *Leishmania* are able to suppress host responses to promote survival and infection establishment. Analysis of cDNAs isolated from dissected sand flies *Phlebotomus papatasi* [9] and *P. perniciosus* [10] midguts revealed that several transcripts which are enriched after receipt of a blood meal are depleted when flies are fed blood containing *Leishmania*. These included digestive proteases, such as trypsins, as well as peritrophins, which are chitin-binding components of the peritrophic matrix that serves as a temporary barrier to leishmania [11]. Moreover, the influence of *Leishmania* infection on physiological responses (oviposition and digestion) or traits (longevity) are far from dramatic [12].

Recently, we described both the host [11] and parasite [12] transcriptomes in a trypanosomatid–dipteran insect infection model, namely, *Drosophila melanogaster* and its natural trypanosomatid parasite *Herpetomonas muscarum*. We showed that parasite feeding resulted in differential transcription of two nuclear factor-κB (NF-κB) pathways, the Toll and the Immunodeficiency (Imd) signaling pathways, as well as the dual-oxidase (DUOX) pathway and STAT-dependent epithelial stem cell proliferation pathway. We found [12] that the *H. muscarum* transcriptome during infection closely resembled that reported for *Leishmania major* during *Phlebotomus duboscqi* infection [13]. Transcriptional responses in *Drosophila* were detected in whole flies, and so even if *H. muscarum* infection was gastrointestinal, there seemed to be a systemic response involving the gut, the fat body and several secreted neuropeptides, all of which were presumably important in inter-tissue communication [11].

Given these findings, we believed a comparison of the *Drosophila* systemic transcriptional responses to those of sand flies during infection would provide valuable knowledge. Common transcriptomic signatures between the two dipteran insects would indicate an evolutionarily conserved response to trypanosomatid immune challenge. Such responses would be of great interest in terms of developing broad-spectrum transmission blocking strategies for trypanosomatid diseases. Conversely, different responses would imply clade-specific host–parasite interactions, with the possibility of potential suppression of the responses described above. However, no comprehensive data were available on the systemic response of the sand fly to *Leishmania*. Therefore, we sought to add to the body of work already available for sand fly transcriptomic responses to trypanosomatid infection using next generation sequencing (RNA-seq) in whole flies.

**Fig. 1:**
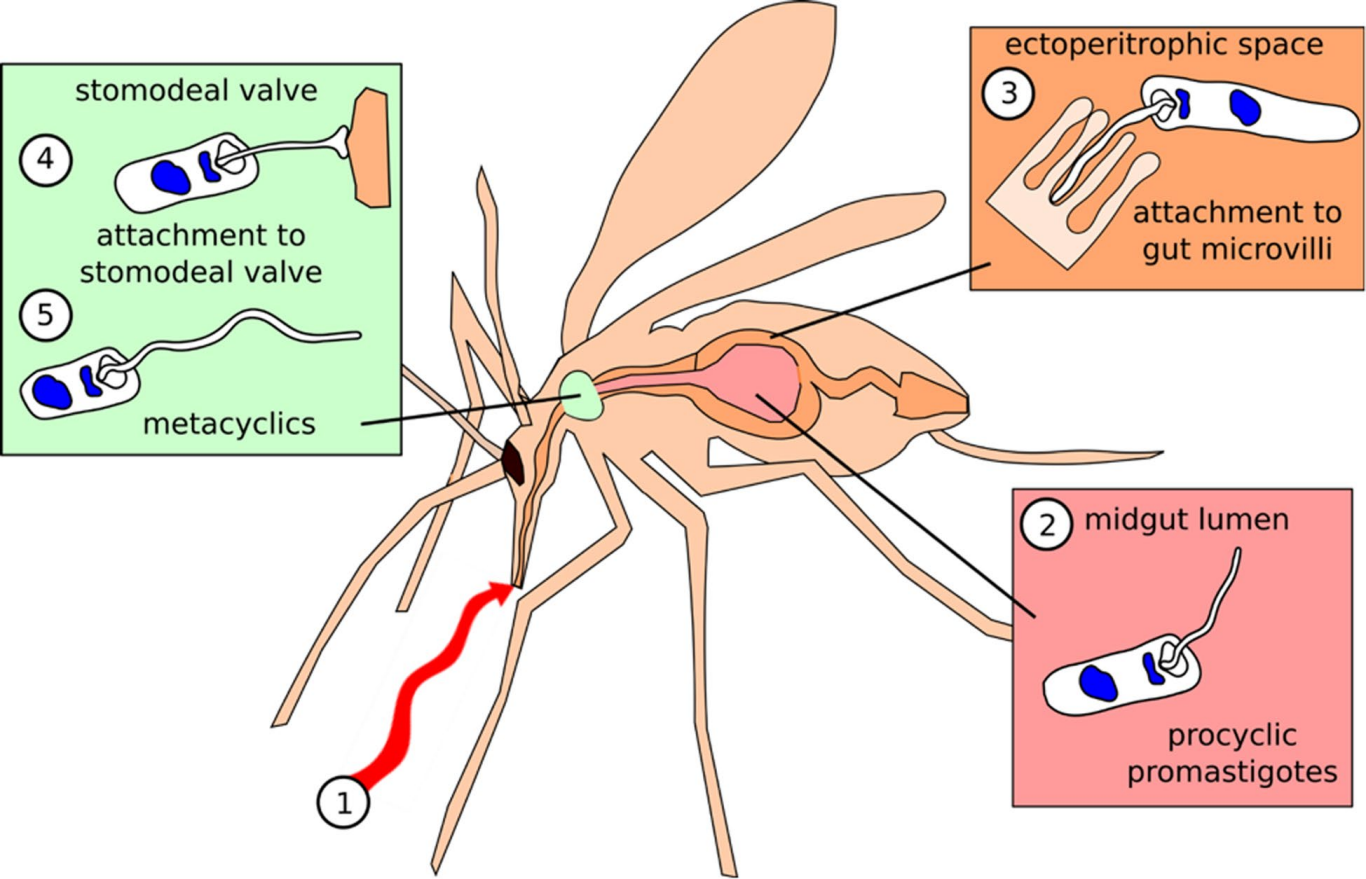
Schematic of the three major *Leishmania* stages in sand flies. Shortly after ingestion (*1*, red arrow) of the blood meal, promastigotes are localized in the midgut lumen, in the blood-meal bolus surrounded by peritrophic matrix (*2*; 1 day post blood meal [PBM]). *Leishmania* wait until the peritrophic matrix is broken down, and then at the end of the digestive process they enter the endoperitrophic space and attach to the epithelial wall (*3*; > 4 days PBM). Finally, where parasites have migrated anteriorly to the thoracic midgut, the stomodeal valve of the fly and the human-infective metacyclic forms differentiate from the earlier stages (*4*,* 5*; > 9 days PBM).

Here, we describe the transcriptome of sand fly *P. papatasi* at three time points corresponding to important stages of trypanosomatid infection: 1 day post blood meal (PBM); following blood-meal digestion and when parasites can be found attached to the midgut epithelium (4 days PBM); when parasites have migrated anteriorly in the gut and are found in the thoracic midgut and the stomodeal valve of the fly (9 days PBM; Fig. 1) [14]. Infections were conducted in the context of both permissive (*Leishmania major*) and refractory (*Leishmania donovani*) infections, as well as with monoxenous (infects only insects) trypanosomatid *Herpetomonas muscarum,* which is not a natural parasite of sand flies. Using this strategy, we hoped to identify host transcriptional signatures associated with permissive and refractory infection outcomes, in addition to identifying evolutionarily conserved host responses as described above.

Our results indicate that there is very little difference between the transcriptomes of flies fed an infected blood meal and those fed a non-infected blood meal. Comparison of early and late time points of blood-only fed flies showed the transcription of genes from several immune pathways—including the Imd, Toll and JAK-STAT signaling pathways. Activation of these responses despite the absence of parasites in the meal may be a pro-active strategy by the sand flies to prevent infection.

## Materials and methods

### *Phlebotomus papatasi* maintenance

A laboratory colony of *P. papatasi* (originating from Turkey) was maintained in the insectary of the Charles University in Prague under standard conditions (26 °C, 60–70% humidity, 14/10 h ligh/dark photoperiod; fed on 50% sucrose) as described previously [15].

### Trypanosomatid maintenance

*Leishmania donovani* (MHOM/ET/2010/GR374), *L. major* LV561 (LRC-L137; MHOM/IL/1967/Jericho-II) and *Herpetomonas muscarum* [11] were cultured in M199 medium (Sigma-Aldrich, St. Louis, MO, USA) containing 10% heat-inactivated foetal bovine serum (FBS; Gibco, Thermo Fisher Scientific, Waltham, MA, USA) supplemented with 1% Basal Medium Eagle vitamins (Sigma-Aldrich), 2% sterile urine, 250 μg/ml amikacin (Amikin; Bristol-Myers Squibb, Princeton Pike, NJ, USA) at 23 °C (*L. donovani*, *L. major*) or 28 °C (*H. muscarum*).

### *Phlebotomus papatasi* infections

*Leishmania* and *H. muscarum* promastigotes from log-phase cultures (3–4 days post inoculation) were resuspended in defibrinated and heat-inactivated rabbit blood (LabMediaServis, Jaroměř, Czech Republic) at concentration of 1 × 10^6^ promastigotes/ml, which corresponds to 500–1000 promastigotes per *P. papatasi* female [16]. Sand fly females (5–9 days old) were infected by feeding on the suspension through a chick-skin membrane (BIOPHARM, Žďár, Czech Republic). Engorged sand flies were maintained under the same conditions as the colony. Each batch was left to develop an infection for 1, 4 or 9 days PBM. In terms of age, on the ninth day PBM sand flies would have been 14–18 days old. Across infections, each batch that we compared had the same age unless we were comparing batches with the same infection. There was no uninfected control for the age of the flies because our question was the difference between blood meal (our uninfected control) and infected blood meal. Age-related signatures would have been contained within the non-infected blood meal. Since systemic sand fly immunity does not become constitutively active during early healthy aging in the absence of infection, we attributed the observed immune activity to receiving a blood meal (infected or not).

### RNA extraction and sequencing

#### Transcriptomic libraries

Poly-A mRNA was purified from total RNA using oligodT magnetic beads, and strand-specific indexed libraries were prepared using the KAPA Stranded RNA-Seq kit followed by ten cycles of amplification using KAPA HiFi DNA polymerase (KAPA Biosystems, Wilmington, MA, USA). Libraries were quantified and pooled based on post-PCR analysis (Agilent bioanalyzer; Agilent Technologies, Santa Clara, CA, USA), and 75-bp paired-end reads were generated on the Illumina HiSeq v4 sequencing system (Illumina Incs., San Diego, CA, USA) following the manufacturer’s standard sequencing protocols. All raw sequencing reads are available on the European Nucleotide Archive under study accession number PRJEB35592.

#### Read mapping and differential expression analysis

Reads were mapped to the *P. papatasi* genome (Ppapl1 v1; Vectorbase) [17] using the HISAT2 alignment program2 [18]. Those reads that mapped uniquely and in their proper pair were extracted and used to assemble transcripts *de novo* with the Cufflinks tool (Tuxedo suite) [19]. These newly assembled transcripts were combined with the VectorBase transcript assembly to create a new set of transcripts using the CuffMerge Tool. Both the sequences of the assembled transcripts and the new annotation file (.gtf) are given in the Electronic Supplementary Material (ESM) data files. Reads were then counted against the transcripts generated by the Cufflinks tool using featureCounts [20]. The counts data for the (two) technical replicates for each sample (= each sample was sequenced twice) were collapsed prior importing into R for differential expression analysis (pairwise Wald tests) in DESeq2 [21]. A DESeq dataset was produced from the counts’ matrix using the inbuilt function using the experiment design: design = ~ Batch + Day + Condition. Pairwise Wald tests were used to identified differential transcript levels between samples. Reported *P* values are adjusted using the Benjamin–Hochberg method (the default in DESeq) to correct for multiple testing.

Analysis performed to conclude that several transcripts lacked conserved domains or that novel transcripts presented conserved domains used the NCBI conserved domains search tool (https://www.ncbi.nlm.nih.gov/Structure/cdd/wrpsb.cgi) and the CDD v3.18 database. This is a superset including NCBI-curated domains and data imported from the Pfam, SMART, COG, PRK and TIGRFAM databases.

## Results and discussion

### Read mapping, *de novo* transcript assembly and differential expression analysis

Sand flies aged 5 to 9 days were fed blood or parasite-containing blood, resulting in four treatment groups of *Phlebotomus papatasi* females: (i) blood meal only, (ii) blood meal + *L.* major, (iii) blood meal + *L. donovani* and (iv) blood meal + *Herpetomonas muscarum.* Flies were then left to develop an infection or digest their non-infected blood meal for 1, 4 or 9 days (actual age = 18–19 days on ninth day PBM). Across infections, flies were compared at the same time point (same age) unless flies with the same infection were being compared.

RNA was purified from whole sand flies at 1, 4 and 9 days PBM, with the aim to identify any systemic responses over and above tissue-specific signals. Each time point analyzed included 20 flies; thus, 3 (time points) × 20 sand flies were processed per treatment group. This experiment was independently repeated three times for each treatment group so that ultimately 3 (replicates) × 60 (sand flies) =180 sand flies were processed for each treatment group in all three experiments. The goal of our experiment was to determine whether there was a difference between blood meal* versus* infected blood meal. Therefore, the non-infected blood meal served as the control.

As expected, only *L. major* was able to establish infection following digestion of the blood meal and defecation at day 4 PBM (Additional file 1: Figure S1). Of these infections, 95% developed into severe *L. major* infections at 8 days PBM, while the infections by the other two parasites were cleared (Additional file 1: Figure S1). At this time point (4 days PBM), *L. major* parasites were found in both the anterior and thoracic midgut as well as at the stomodeal valve (SV) (Additional file 2: Figure S2). In mature *Leishmania* infections, the SV is forced open and becomes blocked with parasites embedded in the promastigote secretory gel, a viscous mixture of phosphoglycans secreted by the parasites. This opening of the SV is essential for colonization of the foregut and transmission by regurgitation [12].

The resulting reads were sequenced and mapped against the *P. papatasi* genome (Ppapl1, Vectorbase) [17]. The number of reads generated per sample ranged from 1.08 to 12.05 million reads with 69.7–79.3% of these mapping to the *P. papatasi* genome in each sample (Additional file 3: Table S1). Upon visual inspection of read mapping using Integrative Genomes Viewer [22], it appeared that > 20% of reads were mapping to regions that lacked annotated features. To include these potentially novel genes in our analysis we assembled *de novo* concordantly mapped read pairs (from all samples) into 16,025 transcripts. The assembled transcripts were then merged with the existing annotation of 11,834 transcripts to give a final set of 18,592 unique transcripts (see ESM data files). This represents approximately 97.2 Mb of *P. papatasi* transcriptome with an average transcript length of 4190 bp. All reads were then counted against the final set of transcripts for differential expression analysis.

**Fig. 2:**
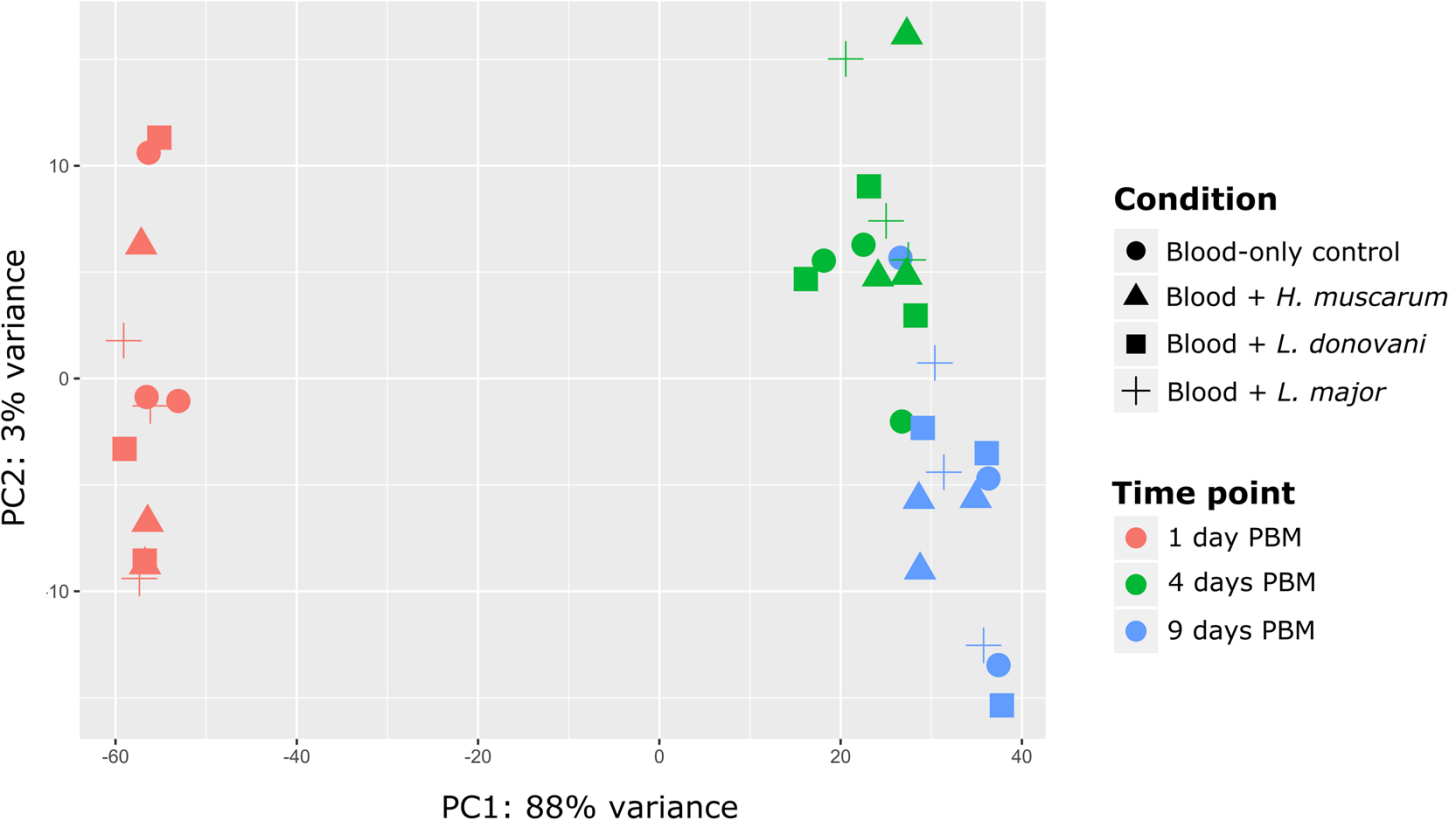
Principal component analysis showing that time was the major source of variation in the experiments with the trypanosomatids *Herpetomonas muscarum*, *Leishmania donovani* and *L. major*—and not infection status (condition).* PC1*,* PC2* Principal components 1 and 2, respectively

Principal component analysis (PCA) showed a high degree of difference between the fly transcriptomes at day 1 PBM and those at day 4 or 9 PBM (Fig. 2), with transcriptomes from days 4 and 9 PBM appearing to be similar. It was also noted that samples did not clearly group in accordance with trypanosomatid feeding status.

### Differential expression associated with trypanosomatid presence in the blood meal

**Fig. 3:**
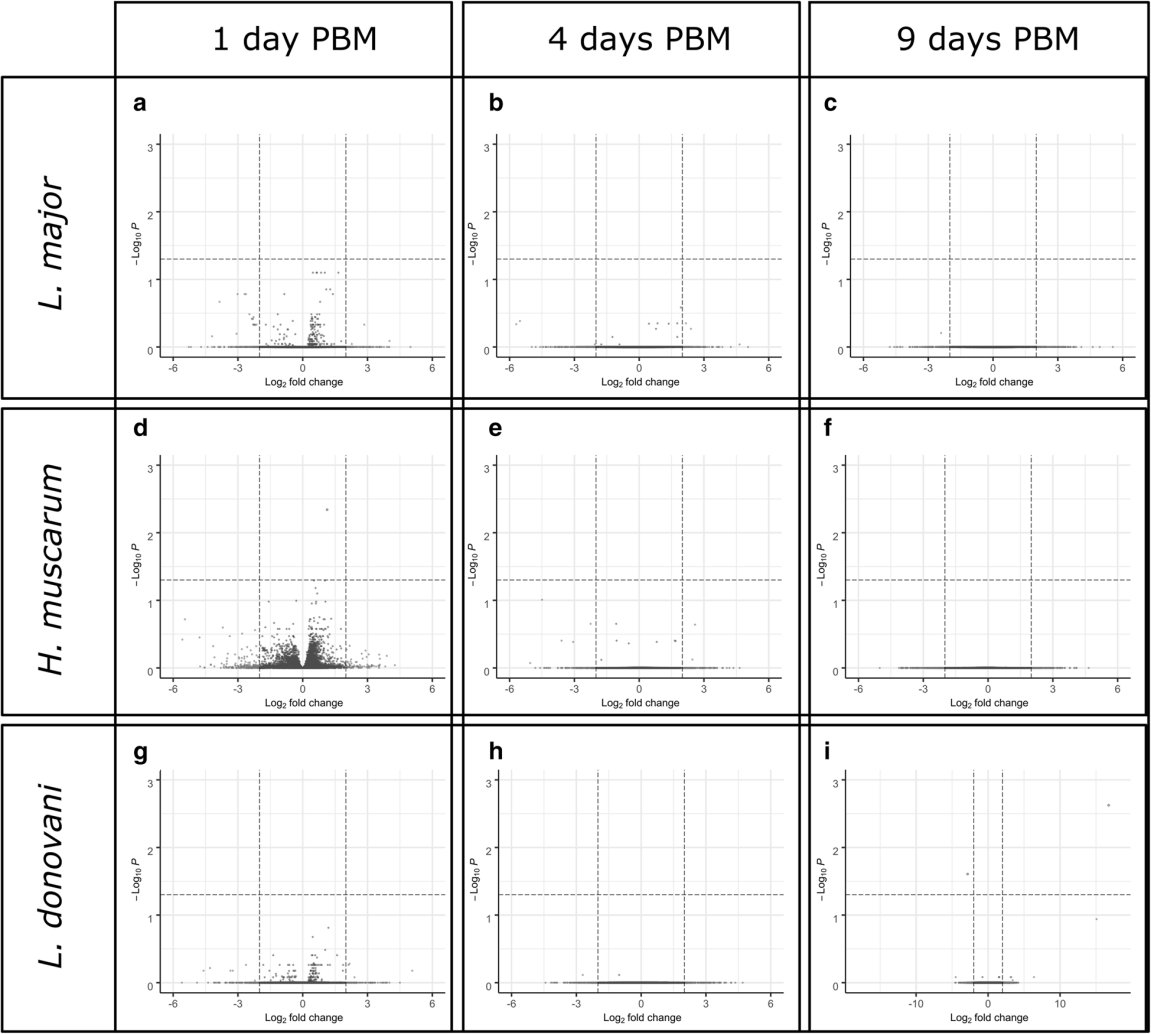
Volcano plots of statistical significance against log2-fold changes in transcript abundance. Blood-fed *Phlebotomus papatasi* sand flies were compared to *P. papatasi* fed *L. major* ( **a**–**c**), *H. muscarum* (**d**–**f**) and *L. donovani* (**g**–**i**). Dashed lines indicate the log2 fold change = − 2/2 and *P* = 0.05 thresholds. Transcripts in red dots exceed the statistical significance threshold. Green dots indicate transcripts which exceeded the fold-change thresholds but were not statistically significantly different between the two feeding conditions. Gray dots indicate transcripts which meet neither of the statistical or fold-change thresholds. Analysis was performed in DESeq2 package for R and visualized using the EnhancedVolcano function

A few differentially expressed transcripts specifically associated with trypanosomatids were present in the blood meal (Additional file 4: Table S2). We found no significant difference in transcript abundance between blood-fed and *L. major*-fed flies at any time point. Furthermore, we found in excess of 12,000 genes for which we rejected the hypothesis that expression had changed by twofold or more in either direction in pairwise comparisons (Wald test) between blood-only fed and blood+trypanosomatid-fed flies (Fig. 3; Table 1).

**Table 1:** Number of transcripts showing no significant change in expression by twofold or more in either direction between the blood-fed and trypanosomatid-fed sand fly *Plebotomus papatasi*

*vs.*Versus	Number of genes showing no significant change in expression by twofold or more in either direction (*P* < 0.05)								
	*Leishmania major*-fed flies			*Leishmania donovani*-fed flies			*Herpetomonas muscarum*-fed flies		
	1 day PBM	4 days PBM	9 days PBM	1 day PBM	4 days PBM	9 days PBM	1 day PBM	4 days PBM	9 days PBM
Blood-fed 1 day PBM	12,586			12,797			11,957		
Blood-fed 4 days PBM		12,188			12,597			12,356	
Blood-fed 9 days PBM			12,762			12,731			12,634

PBM, Post blood meal

Differential abundance for some transcripts, however, was observed after *H. muscarum* and* L. donovani* feeding compared to blood-only feeding on control flies (Fig. 3d, g, respectively). There were significantly fewer transcripts for the gene *PPAI009043*, an orthologue to the *D. melanogaster* signalling protein Rho GTPase activating protein at 54D (RhoGAP54D), in flies fed *H. muscarum* than in the blood-fed controls at day 1 PBM (log2 fold-change 1.13, *P-*adj = 0.005). The *Aedes aegypti* and *Anopheles gambiae* *RhoGAP54D* orthologues are upregulated in blood-fed mosquitos compared to sugar-fed controls [23, 24]. Given this and that we did not see this response after *Leishmania* feeding, we suggest that this transcriptomic response may be *H. muscarum* specific. The biological significance of reduced RhoGAP54D transcription in this context remains unclear; however, the protein is linked to epithelial morphogenesis during *Drosophila* development [25] and so may also play a role in the mature insect gut.

In *L. donovani*-fed flies there were significantly fewer transcripts for the putative transporter TrpA1 (PPAI004036; log2 fold-change 2.8,* P*-adj = 0.025)* versus* blood-only fed flies at 9 days PBM. TrpA1 is more generally associated with chemo- and thermo-sensing [26, 27] in *Drosophila*; however a study by Du et al. [28] links TrpA1 to the expulsion of food-borne pathogens by increased defecation and the DUOX pathway (discussed in detail later in text). It can be speculated that a reduction in TrpA1 transcripts after *L. donovani* feeding may indicate modification of host defensive pathways to promote survival. We also found significantly more CUFF.12679 transcripts (log2 fold-change 16.8, *P*-adj = 0.0001) in *L. donovani*-fed flies than in blood-only fed flies. This novel transcript lacks conserved domains or sequence similarity to known dipteran gene transcripts.

Similarily, direct comparisons between trypanosomatid infections yielded few differentially expressed transcripts (Additional file 5: Table S3). At day 1 PBM the only differentially expressed transcript between the three infections was that of trypsin 1 (*PPAI010956*; * P*-adj = 0.035) which was twofold enriched in *H. muscarum*-fed flies compared to those fed *L. donovani*.

After defecation at around 4 days PBM, it is thought only parasites able to establish in the ectoperitrophic space persisted to develop mature infection [16]. Despite the differences in the infection outcome reported in laboratory infections across the three trypanosomatids [14], there were few differences in the host transcriptome at this critical time point. Two transcripts were found to be significantly differentially abundant: one corresponding to the *PPAI000999* gene and the other a novel transcript CUFF.14170. Both transcripts were found at significantly higher levels (*P*-adj = 0.04 and 2.27E-09, repectively; log2 fold-changes 4 and 18, respectively) in *H. muscarum*-fed flies compared to those fed *L. donovani*. *PPAI000999* encodes a protein predicted to bind to chitin (GO:0006030, GO:0008061 and smart00494). The novel transcript CUFF.14170 has no known conserved domains, and BLAST searches against dipteran sequences did not yield any significant hits.

The most variation between the three infections was found at 9 days PBM, where six transcripts were found to be differentially expressed between *Leishmania*-fed and *H. muscarum*-fed flies. Compared to sand flies fed *H. muscarum*, those fed *L. donovani* had significantly more transcripts for *TrpA1* (*PPAI004036*) and significantly fewer for the putative zinc metalloprotease PPAI010164 and novel transcript CUFF.12679. Sand flies fed *L. major* had significantly more transcripts for the hypothetical protein PPAI002947. Additionally, feeding with *H. muscarum* resulted in significantly more CUFF.14170 transcripts, a novel transcript identified in this study which lacks conserved domains, than both *Leishmania* infections (*P*-adj = 1.79E−09).

Overall, the above observations suggest that blood-feeding status is the major source of transcriptional variation in these sand flies—and not trypanosomatid infection. As such, we further investigated transcriptomic changes after blood-feeding alone in *P. papatasi*.

### The* P. papatasi* transcriptome after blood-feeding

Shortly after blood-feeding there were significant changes in transcription that may be a universal response to a blood meal. The transcriptomes at day 1 PBM appeared to be very different to those at 4 (and 9) days PBM, with 12,289 significantly differentially regulated transcripts (Additional file 6: Table S4). However, after defecation of the blood-meal remnants, the transcriptome was comparatively stable with 264 differentially regulated transcripts (4* vs* 9 days PBM; Additional file 7: Table S5). Due to the large number of differentially expressed transcripts highlighted by these comparisons we first investigated transcripts whose log2 fold-change was > 4 in either direction between time points. From this subset we were able to focus our analysis on a number of key genes and pathways that are discussed in subsequent sections (Additional file 8: Table S6; Additional file 9: Table S7).

### Early transcriptomic responses to ingestion of blood meal are related to digestion, metabolism and immunity

Of the 217 transcripts differentially regulated by > 4-fold between 1 and 4 days PBM, 197 transcripts were found to be comparatively enriched at day 1 PBM and 20 were comparatively enriched at day 4 PBM; 98 of these transcripts did not contain known conserved domains.

Transcripts for putative and known trypsins were one of the most highly represented groups differentially regulated between day 1 and day 4 PBM. We observed upregulation of nine transcripts for putative trypsins and chymotrypsins—including the previously characterized chymotrypsins 1 (PPAI010833), chymotrypsin 3 (PPAI005023) and trypsin 4 (PPAI010456) [8, 29, 30]. We also observed upregulation of transcripts that may represent novel trypsins based on conserved domains and their similarity to other dipteran trypsin/chymotrypsin sequences, as they are not included in the current genome annotation (Ppap v1) [17] (CUFF.11666, CUFF.9493, CUFF.6542 and the chymotrypsins CUFF.15058, CUFF.16005, CUFF.15086, CUFF.14587, CUFF.12454). In contrast, the transcript putatively encoding for trypsin 1 (PPAI010956) was shown to be enriched at day 4 PBM compared to the earlier time point. The roles of trypsin and chymotrypsin-like serine proteases during blood digestion in hematophagous insects are well characterized, with expression levels varying according to the type of blood meal and the time since the last blood meal. Our findings agree with those reported previously showing upregulation of trypsins 3/4 and chymotrypsin 1 in response to the blood meal, as well as the decrease of trypsin 1 [30].

In addition to the trypsins themselves, five transcripts whose products are predicted to contain trypsin inhibitor-like domains (PPAI003932, PPAI000270, PPAI000272, PPAI000274, PPAI003557) were also comparatively enriched at day 1 PBM (*vs* day 4 PBM). It is possible that the corresponding proteins play roles in the regulation of the trypsin 1 as well as other trypsins (*e.g.* trypsin 2), reported to be downregulated after blood-feeding [30].

Several transcripts encoding for proteins with predicted serine protease/proteolytic activity, the sequences of which do not resemble trypsins/chymotrypsins, were also comparatively enriched at day 1 PBM. These included two known genes (*PPAI009419, PPAI009871*) and three novel transcripts (CUFF.6132, CUFF.6133, CUFF.16132). Serine proteases are implicated in several other cellular processes, including innate immune signaling—notably in Toll pathway activation [31]—and the melanization response [32]. The predicted protein for PPAI009419 shares approximately 51% identity with the *Culex quinquefasciatus* CLIPA15 (also known as masquerade) across its sequence. CLIPA proteases interact with and regulate other CLIPs and with the prophenoloxidases (PPO) involved in melanization [33, 34]. This response produces reactive quinones which then polymerize to form the dark insoluble pigment melanin. These molecules can encapsulate and isolate invading pathogens or toxic compounds. They also locally generate high local levels of cytotoxic reactive oxygen species (ROS) and prevent gas diffusion, starving the invading pathogen of oxygen. In addition to the putative CLIPA transcript, four pro-phenoloxidase transcripts were upregulated in early blood meal (*PPO1*: *PPAI008831, PPAI010450*; *PPO2*: *PPAI012836, PPAI012835*). These zymogens are the rate-limiting enzymes in the production of melanin. PPO1/2 and CLIPA15 were also found to be upregulated immediately after blood-feeding in *Anopheles gambiae* [24], suggesting that this is a conserved response to blood-feeding in dipterans.

We also observed differential transcription of another group of proteins reported to play vital roles in protection against invading pathogens—peritrophins. These core components of the peritrophic matrix have been shown to be a major barrier against infection establishment. Knockdown of Peritrophin 1 (Per1) in *P. papatasi* results in an approximately 40% increase in *Leishmania major* load at 48 h after parasite ingestion [35]. In our study, *Per1 *transcripts were highly enriched at day 1 PBM (*vs* day 4 PBM) with a log2 fold-change of 9.96. Of the 32 annotated peritrophins in the *P. papatasi* genome, 14 were found to be significantly differentially regulated between days 1 and 4 PBM (Table 2). The majority of transcripts were comparatively enriched at day 1 PBM; however Per2 and Per28 transcripts were more abundant at later time points. Ramalho-Ortigão et al. [10] showed that *P. papatasi* *Per1* transcripts were enriched in flies fed a blood meal compared to a sugar meal, while *peritrophin 2* (*Per2*) transcripts were comparatively depleted in blood-fed flies. Additionally, the group showed that transcripts for both *Per1* and *Per2 *were depleted in *L. major*-infected flies compared to those fed only blood [29]. Our data largely agree with these findings. However, in our study transcript levels were not statistically significantly different between trypanosomatid-fed sand flies and those fed blood only—although we did observe fewer transcripts for *Per2* (*PPAI009723*) in trypanosomatid-fed flies at day 4 PBM (Additional file 10: Figure S3). Other than *Per2*, the patterns in peritrophin transcript abundance for trypanosomatid-fed flies resembled those of the blood-fed controls.

**Table 2:** *P. papatasi* peritrophins were significantly differentially regulated between 1 and 4 days post blood meal

Gene name	Gene ID	Log2 fold-change	*P* value (Benjamini-Hochberg adjusted)
Per1	PPAI009353	9.97	2.94E-30
Per26	PPAI004431	3.79	1.48E-02
Per6	PPAI001604	3.29	1.61E-07
Per10	PPAI004716	2.33	1.01E-04
Per7	PPAI002253	2.33	1.26E-03
Per12	PPAI001263	2.02	3.92E-02
Per11	PPAI004749	2.01	4.17E-11
Per27	PPAI008214	1.84	1.57E-02
Per13	PPAI004750	1.71	1.39E-02
Per8	PPAI002033	1.58	8.38E-11
Per3	PPAI006556	1.49	2.39E-06
Per4	PPAI006974	0.95	2.43E-02
Per28	PPAI001796	− 1.19	1.05E-03
Per2	PPAI009723	− 2.28	8.53E-07

Positive fold-change values indicate enrichment at 1 day PBM and negative values indicate enrichment at day 4 PBM

Additionally, transcripts for another chitin-binding protein, PPAI000188, were significantly more abundant at 4 days PBM than at day 1 PBM. The sequence of PPAI000188 resembles the *Lutzomyia longipalpis* protein ChiBi (EU124616.1 [36], 84% protein sequence identity). ChiBi has been shown to be enriched in * L. longipalpis* fed with blood containing *L. infantum chagasi* [36]. Its upregulation here in* P. papatasi* in the absence of *Leishmania* may indicate this upregulation is a more general response to a blood meal, rather than an infection-specific response.

In addition to trypsins, transcripts of several other groups of genes associated with digestion and nutrient uptake were differentially regulated PBM. Several transcripts for lipid metabolism-associated genes were found to be upregulated at day 1 PBM. In addition, eight transcripts corresponded to known extracellular carboxylic ester hydrolases (PPAI002323, PPAI003061, PPAI003086, PPAI005115, PPAI005116, PPAI005680, PPAI009133, PPAI008993). Similarly, transcripts for a putative sterol transfer protein (PPAI008838), and two paralogous membrane fatty acid desaturase genes (PPAI008098 and PPAI002108) were shown to be comparatively enriched at day 1 PBM. One transcript, CUFF.7417, does not correspond to a known gene; however the transcript showed strong sequence similarity to the extracellular carboxylic ester hydrolases paralogues PPAI005115 and PPAI005116 mentioned above (90% identity). Additionally, CUFF.7417 is immediately downstream of PPAI005115/6 in the genome and as such we propose this represents a previously unknown paralogue.

Four transcripts coded for proteins with solute carrier domains (cl00456). These transcripts encode for the two paralogous sodium-coupled monocarboxylate transporters (SCMTs; PPAI005125 and PPAI007402) and two putative SCMTs (CUFF.14648 and CUFF.14649). The SCMTs are transmembrane proteins that move molecules with a single carboxylate group, including pyruvate and lactate, across the plasma membrane in a proton-dependent manner; they are associated with the insect midgut brush border [37]

We found two transcripts, CUFF.17209 and CUFF.15972, whose products are predicted to contain the conserved insect allergen-related repeat domain (pfam06757). These transcript sequences also showed similarity to reported cDNAs for *P. papatasi* microvillar proteins MVP1 and 2, respectively (> 89% identity to mRNA sequences). These proteins were also found previously to be upregulated in sand flies upon ingestion of a blood meal compared to sucrose-fed flies [29]. These transcripts could not be assigned to an annotated gene in the current vector base genome (Ppal1) [17]. The function of these proteins is not well understood although they appear to have a conserved signal peptide at the N-terminus and lack transmembrane domains.

Finally, three olfactory (Or57 [PPAI013155], Or99 [PPAI013290] and the putative protein PPAI002404) and a gustatory receptor orthologous to sweet taste receptors of *Drosophila* (Gr9 [PPAI010978]) were upregulated at day 1 PBM compared to later time points. It is likely these sensory receptors were involved in sensing and acquisition of the blood meal and that subsequent decreases in their transcript abundances may indicate these sensors were not required after digestion.

### The transcriptome after defecation of the blood meal is comparatively stable

The two later time points in this study had similar transcriptomic signatures, with only six transcripts comparatively enriched by > 2-fold at 9 days PBM (*vs.* 4 days). These transcripts corresponded to two glutamate receptors (PPAI003634, PPAI008275), apoptosis inhibitor survivin (PPAI002284), two histone methyltransferases (PPAI005539, PPAI005538) and a mucin (PPAI009152). Mucins have been implicated in the interaction with *Leishmania* parasites. Given that several immunity-related transcripts (including peritrophins, mucins and melanization pathway genes) were upregulated, we postulated that a general immune response was triggered upon ingestion of a blood meal and therefore investigated the transcription of the members of the two major innate pathways after a blood meal, namely the Toll and Immunodeficiency (Imd) signaling pathways. Both pathways have been shown to play a role in the response to trypanosomatids [11, 38–42]. We also investigated members of the DUOX and JAK-STAT pathways, both of which have been implicated in the *D. melanogaster–H. muscarum* interaction [11]. Differential regulation statistics for these transcripts can be found in Additional file 11: Table S8.

### Blood ingestion alone is associated with increased innate immune gene transcription

**Fig. 4:**
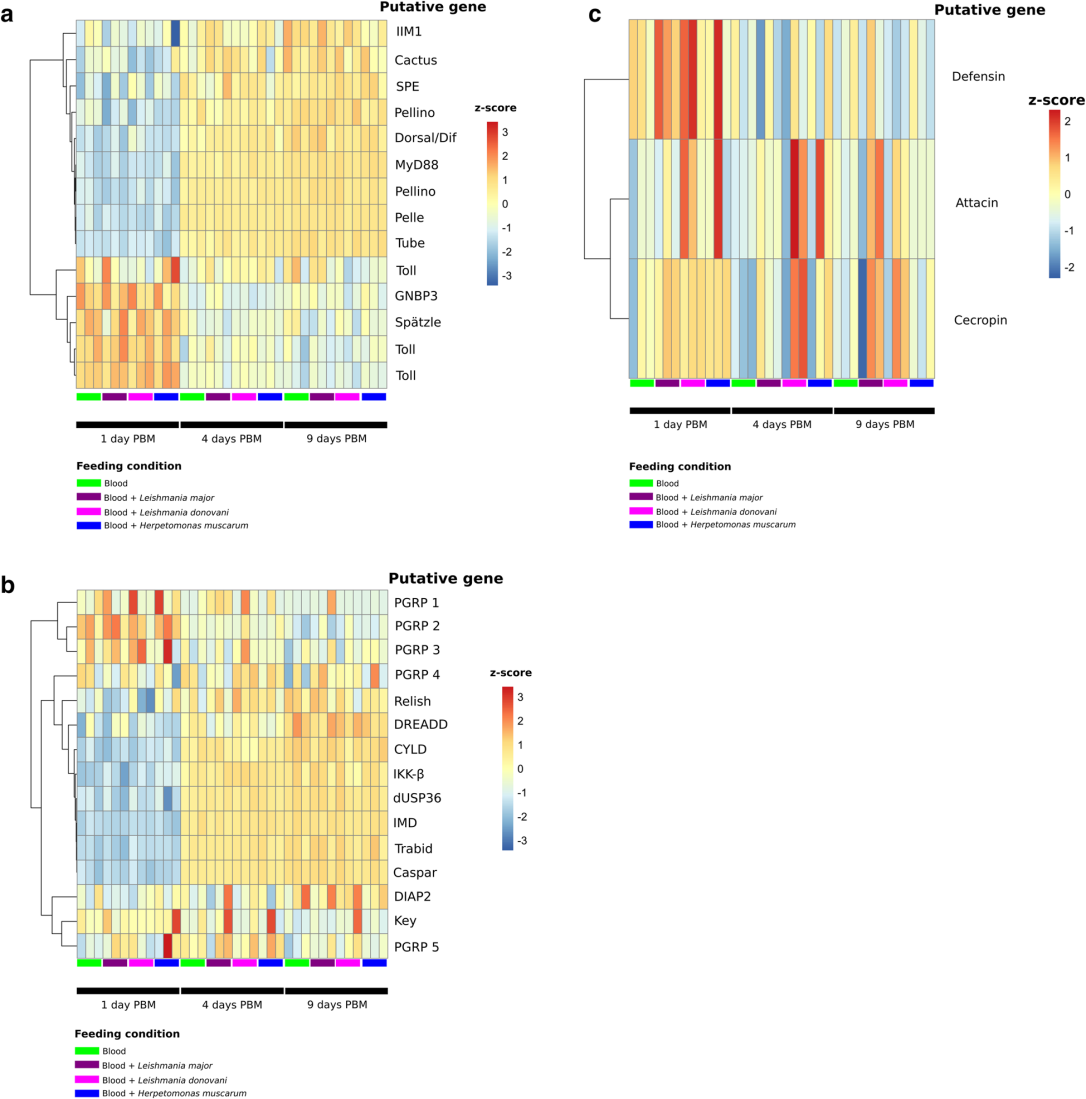
Transcription of genes from the two major innate immune pathways in *P. papatasi* across samples. **a** A heatmap of* Z*-scores (based on log-transformed, normalized counts data) for Toll pathway genes across samples. **b** A heatmap of* Z*-scores (based on log-transformed, normalized counts data) for Immunodeficiency (Imd) pathway genes across samples. **c** A heatmap of* Z*-scores (based on log-transformed, normalized counts data) for anti-microbial peptide genes across samples

In blood-fed flies, transcripts putatively encoding two early Toll pathway genes (*Spätzle* and *GNBP3*) were found to be significantly enriched at day 1 PBM compared to days 4 and 9 PBM (Fig. 4a). One exception was the Spätzle-processing enzyme (SPE), the putative transcript for which was enriched at the latter two time points along with several intracellular Toll pathway components, indicating the possibility of a positive feedback loop back to Spätzle. These trends were broadly consistent in sand flies fed blood only as well as those fed with each of the trypanosomatids. However, only flies fed with blood containing *L. major* or *L. donovani* promastigotes had significantly higher levels of transcripts encoding Toll pathway inhibitor Cactus at day 1 PBM compared to day 4 PBM (> 2-fold, *P*-adj = 0.01 and 5.08E-05, respectively). Cactus transcript abundance was not significantly different between days 1 and 4 PBM in sand flies fed blood or *H. muscarum*.

A similar pattern emerged for the IMD pathway (Fig. 4b). Transcripts for putative peptidoglycan recognition proteins (PGRPs) were more prevalent at day 1 PBM compared to later time points (Fig. 4b). However, only putative PGRP 2 (CUFF.5670) was found to be statistically significantly enriched (2.23-fold) at day 1 PBM (*vs* days 4 and 9 PBM). The transcripts putatively encoding IMD, as well as several other proteins downstream of IMD in the pathway were found to be significantly enriched at 4 and 9 days PBM (*vs* day 1 PBM), including DREDD, TAK1 and IKKβ. We also observed significant enrichment of transcripts putatively encoding negative regulators of the IMD pathway Caspar, dUSP36, Trabid at days 4 and 9 PBM. Interestingly, the IMD transcription factor Relish was not significantly differentially regulated in blood-only-fed flies, while flies fed blood containing *L. major* or *L. donovani* promastigotes showed enrichment of putative Relish transcripts at day 1 PBM compared to 4 and 9 days PBM. As such, while there is overall upregulation of IMD pathway transcription with or without trypanosomatids in the blood meal, there may be important differences in the expression levels of the innate effectors the meal regulates when *Leishmania* are present.

Both Toll and IMD result in the expression of a suite of anti-microbial peptides. Transcripts for these immune effectors were not significantly differentially regulated after blood feeding alone. However, flies fed with blood containing *L. major* or *L. donovani* promastigotes were found to have significantly more transcripts for the AMP defensin at day 1 PBM compared to day 4 PBM (2.3 and 1.75 log2 fold-change, respectively). This was not observed in *H. muscarum*-fed flies. While transcript levels for other anti-microbial peptides did change after trypanosomatid feeding, for example we observed elevated transcript levels for cecropin and attacin in some trypanosomatid infections (Fig. 4c), overall these were not found to be statistically significant changes.

**Fig. 5:**
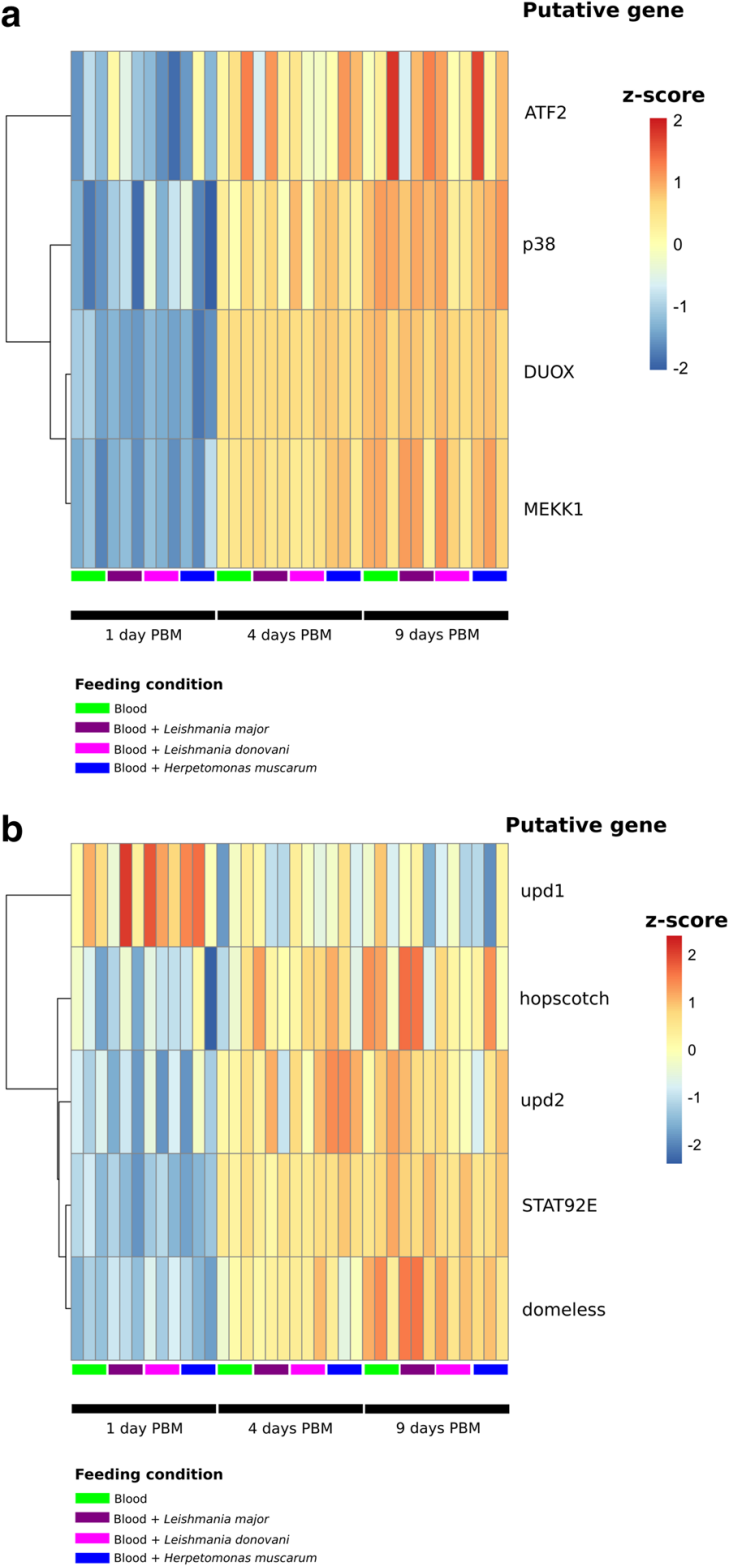
Transcription of genes form the dual-oxidase (DUOX) (**a**) and JAK-STAT (**b**) signalling pathways in *P. papatasi* across samples. Heatmaps of* Z*-scores (based on log-transformed, normalized counts data) across samples

In addition to AMP expression, the IMD pathway can also result in the transcription of the NADPH oxidase, DUOX, through interaction of IMD with MEKK1 [43]. This transmembrane protein is responsible for the production of ROS species in the gut epithelium in response to microbes. We found that the level of DUOX transcripts was significantly higher at days 4 and 9 PBM compared to day 1 PBM in all feeding conditions (log2 fold-change 2.98–3.33; Fig. 5a), with no significant difference in DUOX transcript abundance between 4 and 9 days PBM in any infection condition. Similarly, we saw significant increases in transcripts for genes upstream of DUOX across infection conditions including: the transcription factor ATF2, p38 kinase and MEKK1. As such, induction of DUOX pathway transcription appears to be a generalized response to blood-feeding rather than an infection-specific response.

### The JAK–STAT pathway is also associated with the dipteran response to trypanosomatids

Finally, given the association between the JAK-STAT pathway (Fig. 5b), dipteran gut morphology and immunity [44], particularly in a trypanosomatid infection context [11], we also investigated the transcription of key components of this pathway after blood-feeding. We observed a higher abundance of putative Upd1 transcripts at day 1 PBM compared to later time points; however this change was only shown to be statistically significant for sand flies fed with blood and *L. major* where there was a 2.2-fold enrichment of putative Upd1 transcripts. Furthermore, putative transcripts for the JAK-STAT transcription factor STAT92E were twofold enriched in flies in all infection conditions at the two later time points (*vs* 1 day PBM). We also observed a modest enrichment of transcripts for cytokine Upd2 and the transmembrane receptor Domeless at days 4 and 9 PBM compared to earlier time points (fold-changes 1.19 and 1.74, respectively). The transcription pattern for signaling protein hopscotch resembled that of Domeless; however these transcripts were only found to be statistically significantly enriched in trypanosomatid-fed flies. Together these observations suggested an increase in JAK-STAT signaling a few days after a blood meal in *P. papatasi*. Further work to investigate if this signalling translates to changes in gut homeostasis, such as the increased stem cell proliferation observed in the *Drosophila*–*Herpetomonas* model, will be important. Currently, however, as transcript abundance for STAT92E is enriched in blood only fed controls this response does not appear to be trypanosomatid-specific.

Given the magnitude of the transcriptomic changes associated with blood-feeding alone, and the little variation between blood meals spiked with trypanosomatids that produce very different infections, we speculate that the aforementioned defensive responses are not infection specific. Such a strong response to the blood meal alone is not surprising given the additional stresses associated with the hematophagous habit [45]. The high-risk nutrient attainment method drives the insects to take large volumes of blood at each meal; for example, mosquitoes and tsetse flies expand by up to threefold their pre-meal size after a blood meal [45, 46], which puts enormous mechanical strain on the tissues. In addition to volume of the meal, the content of their meal presents additional problems, such as excess water/ions [47], toxic compounds [48] and bacterial expansion in response to the rich meal [49, 50]. Due to the warm-blooded nature of their victim’s blood temperature, the body temperature of blood-feeding arthropods can rapidly (< 60 s) increase by over 10 °C during their meal [51, 52]. All of these factors must also be overcome even in blood meals which do not contain parasites. Additionally, activation of immune pathways following a blood meal (but in the absence of infection) may be a strategy to ‘pre-emptively’ protect the host against pathogens/toxic compounds which may be present in the newly ingested blood meal. Such anticipatory responses have been reported in other hematophagous insects, including the malaria vector *Anopheles gambiae* [53].

Moreover, it is known that in sand flies, the blood meal is followed by a decrease in overall gut bacterial diversity [54] coupled to an increased abundance of aerobic bacteria [50]. It is possible that these changes may mask any effects from the presence of trypanosomatids. However, there was also no significant difference between day 9 PBM *L. major* and the other day 9 infections. This is important since by day 9 PBM, the blood meal has long been digested and it is only the *L. major* that is left while the other parasites are cleared. This underlines the non-specificity of the *P. papatasi* response and implies that for the sand fly, *L. major* is just another feature in the microbiome landscape. Alternatively, *L. major* establishment in the midgut could mediate a suppression of host responses. Consistent with this, a recent study comparing RNA-seq data generated from the midgut found just a 1% overall difference between sand flies receiving a blood meal and those receiving a *Leishmania-*contaminated blood meal [55]. The limitation of our hypothesis is that we have performed our experiments in whole flies and so it could be that there are infection-specific responses in other tissues. More work is needed to verify that what we see at the organismal level is also the case at the level of immune-competent tissues (gut, hemocytes, fat body).

## Supplementary Information

**Additional file 1: Figure S1.** Time-series of infection intensity (% of infected females) for the three trypanosomatids tested. As expected,* L. major* developed late stage infections while the other two parasites were lost during defecation of blood meal remains.

**Additional file 2: Figure S2.** Time-series of infection localization for the three trypanosomatids used.

**Additional file 3: Table S1.** Read mapping summaries. This table shows the read mapping information for each sample; for example, number of reads, percentage of read mapped, among others.

**Additional file 4: Table S2.** Transcripts associated with trypanosomatid presence in the blood meal. This table shows the fold-changes and differential regulation statistics (including* P* values) for transcripts whose abundance differed between trypanosomatid fed flies and blood-fed control flies.

**Additional file 5: Table S3.** Transcripts associated with specific trypanosomatids in the blood meal. This table shows the fold changes and differential regulation statistics (including* P* values) for transcripts whose abundance differed between trypanosomatid infections.

**Additional file 6: Table S4.** Transcripts significantly differentially regulated between 1 day and 4 days post blood meal (blood-only) in *P. papatasi*.

**Additional file 7: Table S5.** Transcripts significantly differentially regulated between 4 day and 9 days post blood meal (blood-only) in *P. papatasi*.

**Additional file 8: Table S6.** Transcripts of interest which are differentially regulated between 1 day and 4 days post blood meal (blood-only) in *P. papatasi*. This is a streamlined version of Additional file 6: Table S4 showing transcripts of interest discussed in the text.

**Additional file 9: Table S7.** Transcripts of interest which are differentially regulated between 4 days and 9 days post blood meal (blood-only) in *P. papatasi*. This is a streamlined version of Additional file 6: Table S4 showing transcripts of interest discussed in the text.

**Additional file 10: Table S8.** Differential regulation statistics for transcripts of dipteran immune pathways of interest (Toll, Imd, DUOX and JAK-STAT) across samples.* ns* Not significantly differentially regulated.

**Additional file 11: Figure S3.** Log-normalized transcript counts for Peritrophin 2 (*Per2*) in *P. papatasi* throughout infection. Error bars show the standard error of the mean.

## Data Availability

All data generated or analyzed during this study are included in this published article (and its additional information files). Raw data can be found at the European Nucleotide Archive, Project PRJEB35592 (ERP118668).
