# Supplementary figures and images for: The Phlebotomus papatasi systemic transcriptional response to trypanosomatid-contaminated blood does not differ from the non-infected blood meal
Source: Parasit Vectors. 2021 Jan 6;14:15. doi: 10.1186/s13071-020-04498-0 (PMC7789365; doi:10.1186/s13071-020-04498-0)

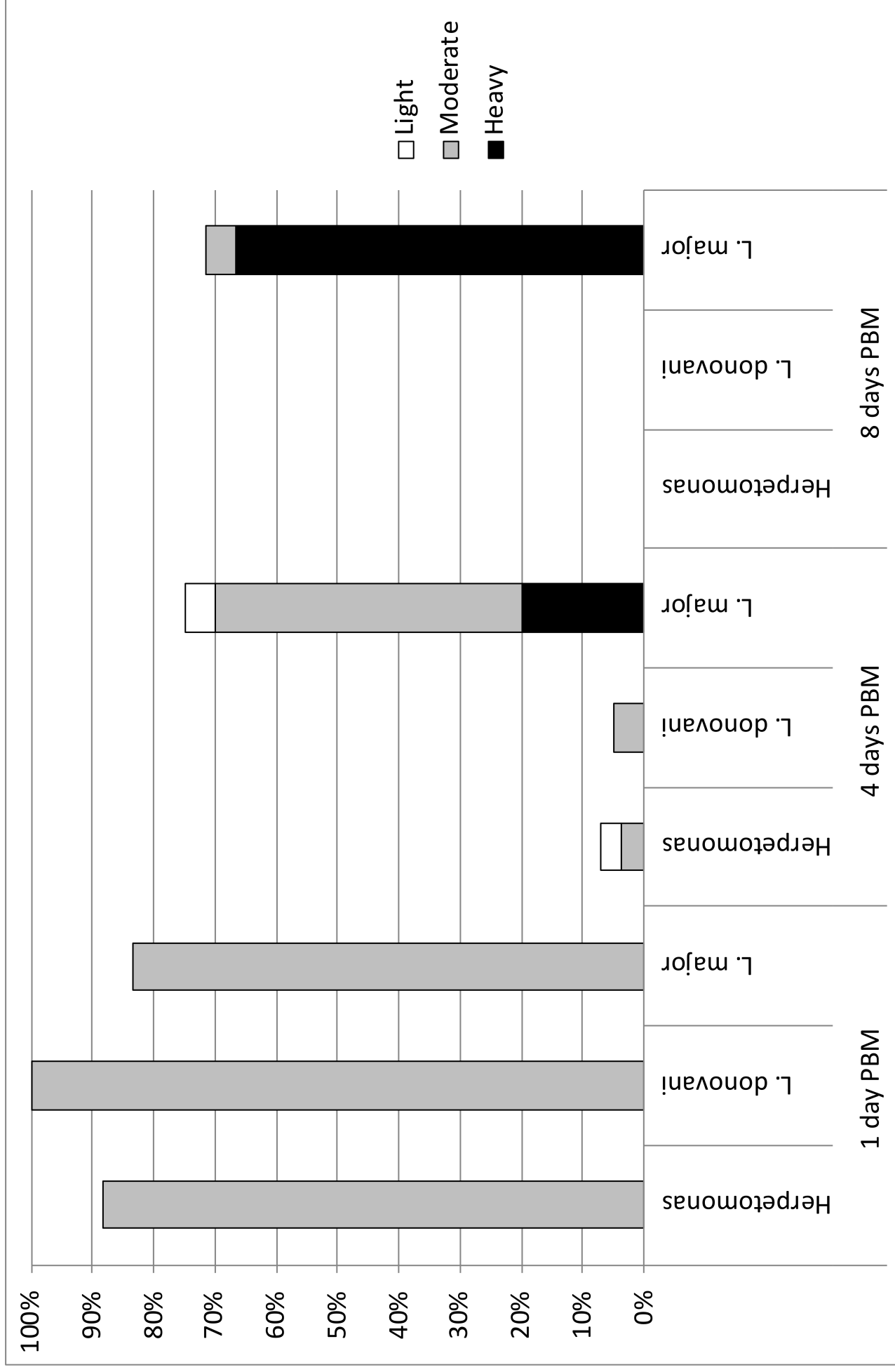

Supplement: Supplementary file 1 — Additional file 1: Figure S1. Time-series of infection intensity (% of infected females) for the three trypanosomatids tested. As expected, L. major developed late stage infections while the other two parasites were lost during defecation of blood meal remains. [file 13071_2020_4498_MOESM1_ESM.pdf]

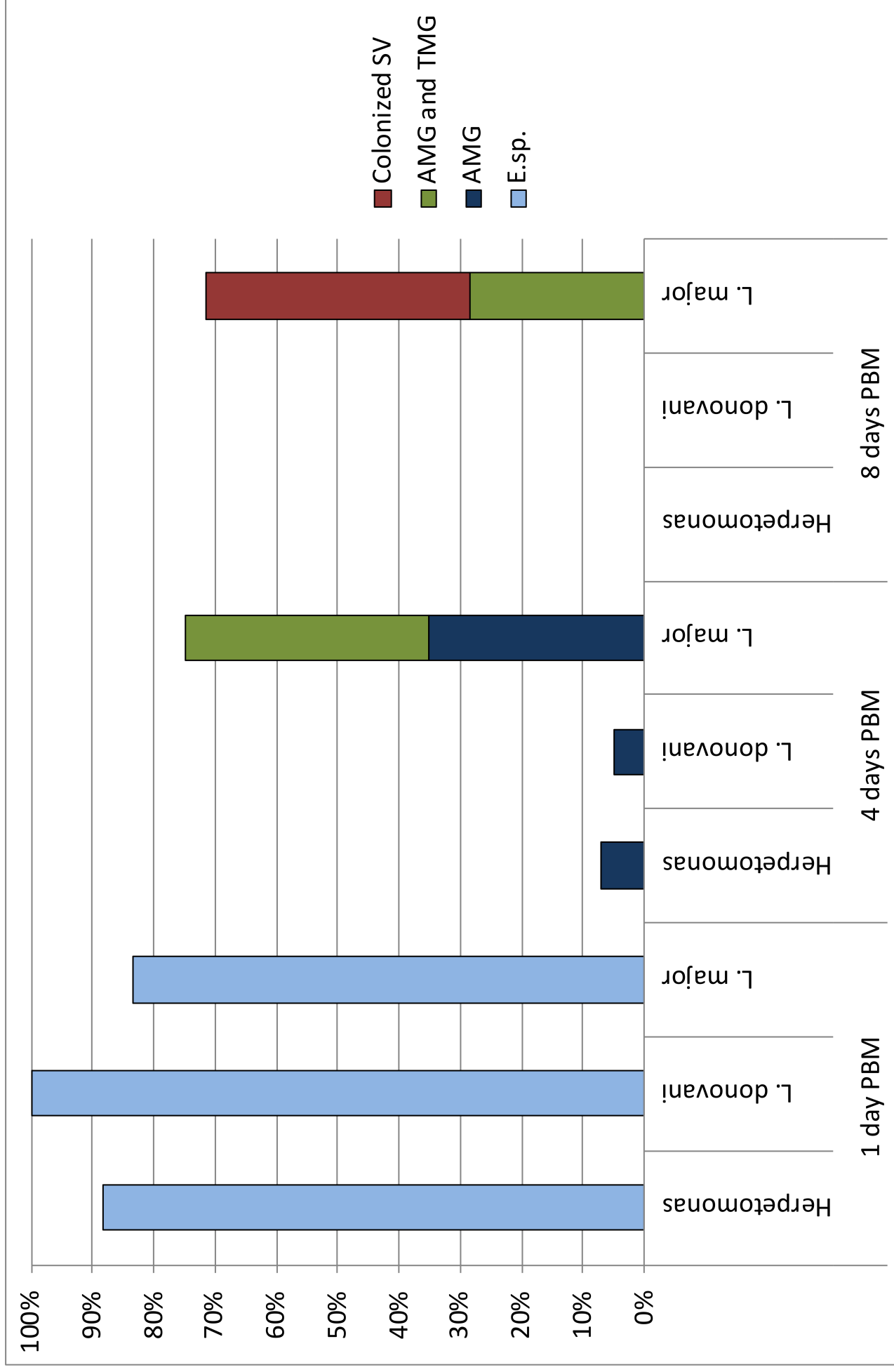

Supplement: Supplementary file 2 — Additional file 2: Figure S2. Time-series of infection localization for the three trypanosomatids used. [file 13071_2020_4498_MOESM2_ESM.pdf]

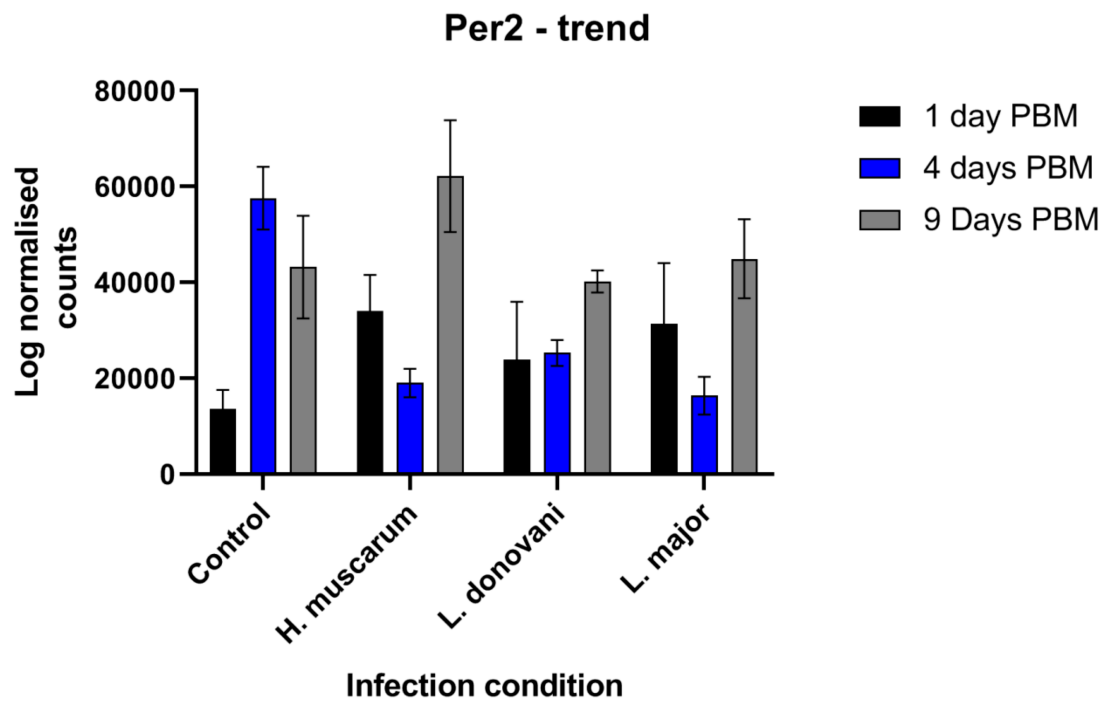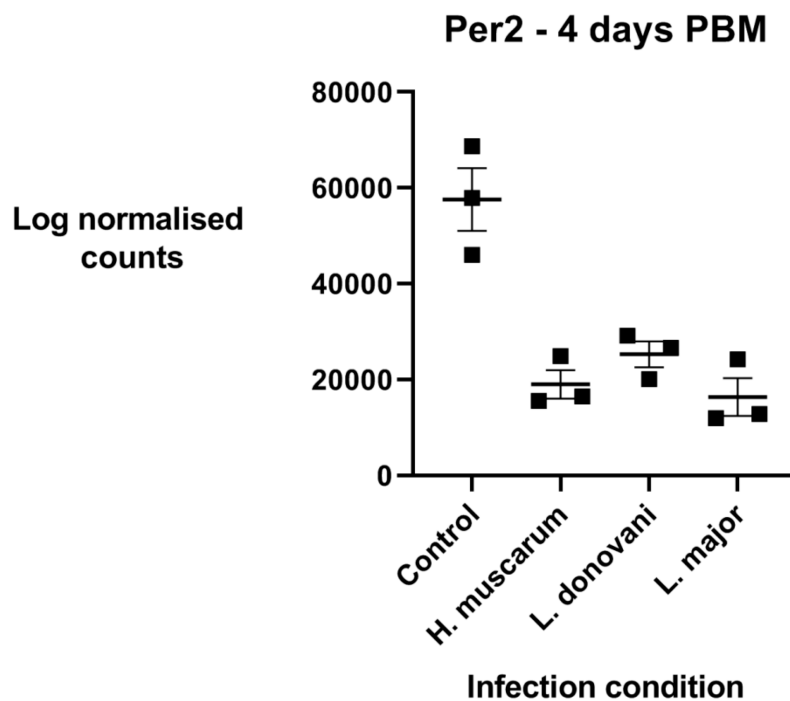

Supplement: Supplementary file 11 — Additional file 11: Figure S3. Log-normalized transcript counts for Peritrophin 2 (Per2) in P. papatasi throughout infection. Error bars show the standard error of the mean. [file 13071_2020_4498_MOESM11_ESM.pdf]
